# Evaluation of an Internally Controlled Multiplex *Tth* Endonuclease Cleavage Loop-Mediated Isothermal Amplification (TEC-LAMP) Assay for the Detection of Bacterial Meningitis Pathogens

**DOI:** 10.3390/ijms19020524

**Published:** 2018-02-09

**Authors:** Owen Higgins, Eoin Clancy, Martin Cormican, Teck Wee Boo, Robert Cunney, Terry J. Smith

**Affiliations:** 1 Molecular Diagnostics Research Group, School of Natural Sciences and National Centre for Biomedical Engineering Science, National University of Ireland, Galway, Ireland; eoin.clancy@nuigalway.ie (E.C.); terry.smith@nuigalway.ie (T.J.S.); 2 School of Medicine, National University of Ireland, Galway and Galway University Hospital, Galway, Ireland; martin.cormican@hse.ie (M.C.); teck.boo@hse.ie (T.W.B.); 3 Irish Meningitis and Sepsis Reference Laboratory, Temple Street Children’s University Hospital, Temple Street, Dublin, Ireland; robert.cunney@hse.ie

**Keywords:** bacterial meningitis, nucleic acid diagnostics, loop-mediated isothermal amplification, multiplex, internal control

## Abstract

Bacterial meningitis infection is a leading global health concern for which rapid and accurate diagnosis is essential to reduce associated morbidity and mortality. Loop-mediated isothermal amplification (LAMP) offers an effective low-cost diagnostic approach; however, multiplex LAMP is difficult to achieve, limiting its application. We have developed novel real-time multiplex LAMP technology, TEC-LAMP, using *Tth* endonuclease IV and a unique LAMP primer/probe. This study evaluates the analytical specificity, limit of detection (LOD) and clinical application of an internally controlled multiplex TEC-LAMP assay for detection of leading bacterial meningitis pathogens: *Streptococcus pneumoniae*, *Neisseria meningitidis* and *Haemophilus influenzae*. Analytical specificities were established by testing 168 bacterial strains, and LODs were determined using Probit analysis. The TEC-LAMP assay was 100% specific, with LODs for *S. pneumoniae*, *N. meningitidis* and *H. influenzae* of 39.5, 17.3 and 25.9 genome copies per reaction, respectively. Clinical performance was evaluated by testing 65 archived PCR-positive samples. Compared to singleplex real-time PCR, the multiplex TEC-LAMP assay demonstrated diagnostic sensitivity and specificity of 92.3% and 100%, respectively. This is the first report of a single-tube internally controlled multiplex LAMP assay for bacterial meningitis pathogen detection, and the first report of *Tth* endonuclease IV incorporation into nucleic acid amplification diagnostic technology.

## 1. Introduction

Bacterial meningitis infection is caused by the hematogenous spread of human commensal bacteria to the central nervous system, resulting in inflammation of the meninges. Infection typically presents with the rapid onset of features including fever, headache, photophobia, nausea, vomiting and neck stiffness [1]. The approximate annual incident rate of bacterial meningitis infection varies, 1–2 cases per 100,000 people with a 25% mortality rate in developed countries, or 1000 cases per 100,000 people with a 50% mortality rate in disease-burdened developing regions [2]. If left untreated, bacterial meningitis is typically fatal, and almost one in every two survivors are left with severe and long-term neurological sequelae [3,4].

*Streptococcus pneumoniae*, *Neisseria meningitidis* and *Haemophilus influenzae* are leading etiological agents of bacterial meningitis infection, with *S. pneumoniae* and *N. meningitidis* responsible for over 80% of all incidents in regions with routine *H. influenzae* vaccination [5,6,7]. Despite this, *H. influenzae* related meningitis remains a serious threat, especially among children under 5 years of age in regions without routine immunization [8]. Although vaccination programs have significantly reduced infection rates associated with these pathogens [1,7], *S. pneumoniae* and *N. meningitidis* vaccines have altered bacterial meningitis epidemiology. The most significant indirect result of these immunization programs has been bacterial serotype or serogroup replacement increasing the incidence of meningitis caused by non-vaccine or non-typeable strains [2].

The early diagnosis and treatment of bacterial meningitis infection significantly reduces associated disease burden [9]. Microscopic examination of cerebrospinal fluid (CSF), and bacterial culture of both CSF and blood samples, are conventional diagnostic approaches [2]. However, culture can take up to 24–72 h, with reduced sensitivity in cases of prior antimicrobial treatment, requiring initial administration of broad-spectrum antibiotics targeting multiple potential causative pathogens. This use of broad-spectrum empiric therapy can expose patients to adverse effects, increase costs and introduce selective pressure favoring the emergence and dissemination of antimicrobial resistance [10]. Delay in diagnosis of bacterial meningitis infection also prolongs the implementation of public health response, essential for reducing disease transmission. Conventional microbiological diagnostics are also inefficient for simultaneous pathogen identification in cases such as: co-infection; differentiation from other infectious diseases such as cryptococcal or tuberculous meningitis; and detection of non-culturable organisms in cases of prior antibiotic treatment [11,12].

Nucleic acid diagnostic technologies offer advantages over conventional methods, with real-time polymerase chain reaction (PCR) being the most commonly employed approach [13]. Compared to traditional diagnostics, real-time PCR provides a faster, higher throughput, closed system technology, improved diagnostic sensitivity and specificity, and the ability to detect non-culturable pathogens [2,14]. In addition, the simultaneous detection of multiple pathogens using multiplex real-time PCR reduces analysis time and reagent cost while also conserving clinical specimens [15]. However, real-time PCR requires expensive thermocycling equipment, making it a less accessible diagnostic technology compared to traditional methods in settings with limited resources.

Loop-mediated isothermal amplification (LAMP), a rapid, highly sensitive and specific, isothermal nucleic acid amplification technique, offers a less expensive alternative to real-time PCR as it does not require thermocycling [16,17]. LAMP has also demonstrated improved speed, diagnostic sensitivity and specificity, and tolerance of inhibitory substances when compared to PCR [18,19,20]. Furthermore, the utility of LAMP as an effective point-of-care (POC) technology for low-resource disease-burdened areas has been demonstrated using inexpensive, handheld thermal instrumentation with basic LED or smartphone monitoring devices, requiring low power sources [21,22,23,24,25].

LAMP is typically performed at 65 °C using forward and reverse inner, outer and loop oligonucleotide primers, in combination with a strand displacement DNA polymerase. Critically, the inner primers contain both sense and antisense target DNA sequences enabling loop structure formation, producing LAMP’s unique rapid self-priming amplification [16,17]. Multiplex LAMP detection however is difficult to achieve as the non-exonuclease activity of the polymerase enzyme is not compatible with standard nucleic acid hydrolysis probes [26]. This is a major limitation for the diagnostic application of LAMP as multiplex capabilities reduce analysis time/cost, conserve clinical specimens and enable incorporation of assay validation internal controls, essential for clinical diagnostics [27].

**Figure 1:**
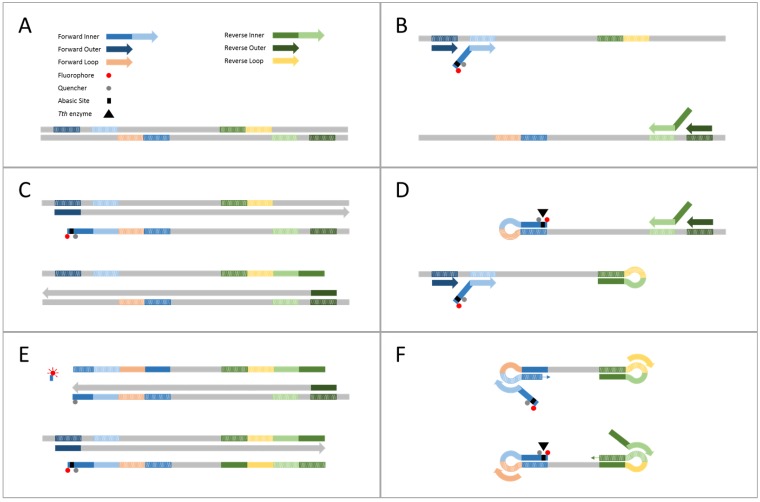
TEC-LAMP mechanism. (**A**) TEC-LAMP oligonucleotide components, *Tth* endonuclease IV enzyme and dsDNA template with oligonucleotide targets highlighted. (**B**) Temperature enabled dsDNA dissociation followed by primer and TEC primer/probe hybridization to corresponding targets. (**C**) Inner primer strand displacement extension, via *Bst* polymerase, forms dsDNA. Outer primer strand displacement extension dissociates this newly formed dsDNA, forming inner primer linked ssDNA. (**D**) The complementary sections of this newly formed inner primer linked ssDNA hybridize, forming loop structures. The abasic site of the TEC primer/probe is now in dsDNA form, and thus, cleaved by the *Tth* endonuclease IV enzyme. TEC primer/probe, inner and outer primers hybridize upstream of the stem loop structures. (**E**): Inner primer strand displacement extension forms dsDNA and displaces the downstream stem loop structures, fully dissociating the TEC primer/probe fluorophore and quencher, producing fluorescence. Outer primer strand displacement extension displaces the newly formed dsDNA, producing inner primer linked ssDNA. (**F**) The complementary sections at each end of the newly formed inner primer linked ssDNA hybridize and form loop structures. These double looped DNA templates are targeted by the TEC primer/probe, inner and loop primers, leading to rapid self-primed exponential amplification with increased cleavage and fluorescence events.

This study introduces *Tth* Endonuclease Cleavage (TEC) LAMP, the first reported single-tube internally controlled multiplex LAMP technology with clinically relevant analytical sensitivity. TEC-LAMP was achieved by incorporating *Tth* endonuclease IV, a thermostable enzyme that cleaves abasic sites present in dsDNA, and a novel TEC primer/probe, with standard LAMP assay conditions. The TEC primer/probe, a modified LAMP forward inner primer incorporating an abasic site flanked by a 5′ fluorophore and an internal thymine coordinated quencher (Table 1), acts as both a real-time monitoring probe and a standard LAMP inner primer. The TEC-LAMP mechanism, outlined in Figure 1, enables real-time monitoring and quantification, simultaneous detection of multiple targets and incorporation of internal amplification control (IAC) validation. We have successfully demonstrated the internally controlled multiplex TEC-LAMP detection of *S. pneumoniae*, *N. meningitidis* and *H. influenzae*, and further evaluated this assay in terms of analytical specificity, limit of detection (LOD) and clinical performance.

## 2. Results

### 2.1. Demonstration of Internally Controlled Multiplex TEC-LAMP Detection

**Figure 2:**
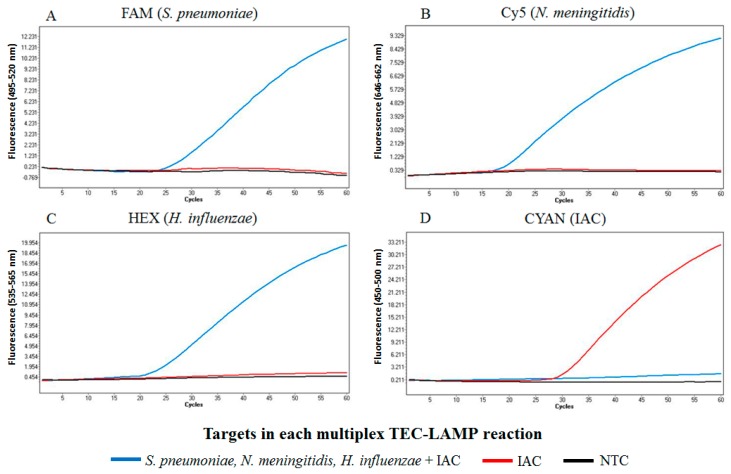
Internally controlled multiplex TEC-LAMP detection. The four graphs show fluorescence recorded in the FAM (**A**), Cy5 (**B**), HEX (**C**) and CYAN (**D**) LightCycler^®^ 480 detection channels, for three TEC-LAMP reactions performed in parallel. These reactions included: (blue) 100 genome copies *S. pneumoniae*, *N. meningitidis* and *H. influenzae* in the presence of 50 copies IAC; (red) no bacterial template in the presence of 50 copies IAC; and (black) a NTC reaction using molecular grade water in place of bacterial or IAC templates. Successful simultaneous detection of all three bacterial targets in the presence of the IAC was observed (blue: **A**–**C**). The two control reactions performed successfully as detection of the IAC in the absence of bacterial target was observed (Red: **D**), and no detection was observed in the NTC reaction (Black: **A**–**D**).

The TEC-LAMP assay successfully demonstrated the internally controlled multiplex detection of 100 genome copies of all three bacterial targets, *S. pneumoniae*, *N. meningitidis* and *H. influenzae*, in the presence of 50 copies IAC template (Figure 2A–C, blue). In this reaction, the IAC template was not detected during the simultaneous co-amplification of the three bacterial targets (Figure 2D, blue). The control reactions performed successfully, with TEC-LAMP detection of 50 copies IAC template in the absence of bacterial template observed (Figure 2D, red) and no amplification observed in the no template control (NTC) reaction (Figure 2A–D, black).

### 2.2. Analytical Specificity and Limit of Detection

The TEC-LAMP assay demonstrated 100% analytical specificity. All inclusivity panel strains were detected in their respective detection channels, *S. pneumoniae* (FAM), *N. meningitidis* (Cy5) and *H. influenzae* (HEX), with none of the exclusivity panel strains detected (Supplemental Table S1). The IAC performed successfully during exclusivity panel testing as it was detected in all events of no bacterial target amplification (Supplemental Table S1). The TEC-LAMP assay LOD, for the separate detection of *S. pneumoniae*, *N. meningitidis* or *H. influenzae* in the presence of 50 copies IAC template, was confirmed with 95% probability using Probit analysis to be 39.5, 17.3 and 25.9 genome copies per reaction, respectively (Supplemental Table S2). The IAC performed successfully during Probit analysis testing as it was detected in all reactions.

### 2.3. Clinical Evaluation

The IMSRL PCR-positive clinical samples that tested positive with the TEC-LAMP assay, were only identified in corresponding detection channels (Supplemental Table S3). Thus, samples positive for a single species were considered negative control samples for the remaining two species. In total, 60/65 positive samples (22/23 *S. pneumoniae*, 22/22 *N. meningitidis* and 16/20 *H. influenzae*), and 0/130 negative samples (0/42 *S. pneumoniae*, 0/43 *N. meningitidis* and 0/45 *H. influenzae*), were successfully identified as such. The 5 IMSRL PCR-positive samples not detected by the TEC-LAMP assay, were subsequently re-confirmed as positive using “in-house” real-time PCR assays, producing similar high Ct-values (>35 Ct) to that of the IMSRL PCR Ct-values. Thus, compared to the IMSRL PCR results, the overall diagnostic sensitivity and specificity of the multiplex TEC-LAMP assay was 92.3% and 100%, respectively. The diagnostic sensitivities for each pathogen were 95.7% (*S. pneumoniae*), 100% (*N. meningitidis*), and 80% (*H. Influenzae*). The IAC performed successfully during clinical sample testing as it was detected in all events of no bacterial target amplification (Supplemental Table S3).

## 3. Discussion

Bacterial meningitis infection is responsible for significant global morbidity and mortality [1]. Rapid and accurate detection of bacterial meningitis pathogens is essential for effective treatment and lowering associated disease burden [9]. This study has evaluated the analytical specificity, limit of detection (LOD) and clinical application of a novel internally controlled multiplex TEC-LAMP assay for the detection of leading bacterial meningitis pathogens: *S. pneumoniae*, *N. meningitidis* and *H. influenzae*.

Analytical specificity of the TEC-LAMP assay was established to be 100%, as all bacterial strains tested were correctly identified in appropriate detection channels (Supplemental Table S1). This confirmed the utility of *SPNA45_01710* and *NMO_1242* genes as effective targets for *S. pneumoniae* and *N. meningitidis*, respectively. Various biomarkers for *S. pneumoniae* (*ply*, *lytA* and Spn9802) and *N. meningitidis* (*ctrA*, *sodC* and *siaD*) have been used for PCR and LAMP assay development [6,15,18,19,28,29,30,31]; however, many of these targets have been identified as inefficient [28,29,32]. We propose that the *SPNA45_01710* gene, and the sequence between bases 450–1800 of the *NMO_1242* gene, are potential novel regions-of-interest for the identification of *S. pneumoniae* and *N. meningitidis*, respectively.

The TEC-LAMP assay LOD, established using Probit analysis (Supplemental Table S2), for *S. pneumoniae*, *N. meningitidis* or *H. influenzae* in the presence of 50 copies IAC template, was determined to be 39.5, 17.3 and 26.5 genome copies per reaction, respectively. Previously reported multiplex PCR assays and singleplex LAMP assays, for *S. pneumoniae*, *N. meningitidis* and *H. influenzae* identification, have shown similar analytical sensitivities of 3–60 and 10–100 genome copies per reaction, respectively [15,18,19,28,29,30,31,33]. Also, the analytical sensitivity of the TEC-LAMP assay is within range of typical target pathogen loads in bacterial meningitis CSF specimens, the standard sample type for bacterial meningitis diagnosis [15].

Clinical application of the TEC-LAMP assay was evaluated by testing 65 anonymized residual samples that had previously tested PCR-positive for either *S. pneumoniae*, *N. meningitidis* or *H. influenzae* (Supplemental Table S3). The diagnostic sensitivity and specificity of the TEC-LAMP assay, defined as the proportion of confirmed-positive and confirmed-negative clinical samples correctly identified as such, was established to be 92.3% and 100%, respectively. Positive results were detected at an approximate average of 20 cycles (Supplemental Table S3) indicating an approximate average time-to-positivity of 20 min per sample. Considering the IMSRL PCR evaluation cycling parameters, 1 cycle of 95 °C for 20 s followed by 45 cycles of 95 °C for 3 s and 60 °C for 20 s, the resulting TEC-LAMP clinical sample time-to-positivity values were significantly faster than the IMSRL PCR results. As rapid bacterial meningitis diagnosis is essential for improved treatment and public health response [9], TEC-LAMP’s faster time-to-detection results compared to real-time PCR highlight its diagnostic utility, especially for disease burdened low resource areas. Clinical performance comparison of the TEC-LAMP assay to the IMSRL PCR assays was chosen as previous studies have identified that comparing nucleic acid diagnostics to less sensitive culture methods can be problematic [11,29]. Successful detection, using “in-house” PCR assays, of the 5 IMSRL PCR-positive samples that were not detected using TEC-LAMP, was possibly due to improved analytical sensitivity of the single-target PCR assays compared to the internally controlled multiple-target TEC-LAMP assay.

This study utilized clinical isolates/samples, from culture or PCR confirmed cases of bacterial meningitis infection, collected by the GUH and IMSRL during routine diagnostic services and in accordance with respective ethical review committee approved protocols. Anonymized residual specimens positive for *S. pneumoniae*, *N. meningitidis* or *H. influenzae* were supplied for TEC-LAMP assay evaluation, and analysis for human DNA was not carried out. Considering this, the Ethics Committee of the National University of Ireland, Galway deemed that ethical approval for the evaluation of these samples was not required.

IAC incorporation into nucleic acid diagnostic assays provides validation by ensuring negative results are due to target absence and not assay performance [27]. The TEC-LAMP detection of IAC template in all events of no bacterial target amplification (Figure 2D, red; Supplemental Tables S1 and S3), successfully validated test results in this study. The events of no IAC detection during specificity testing, clinical testing and the demonstration of multiplex TEC-LAMP detection (Figure 2D, blue), were due to inhibition from amplification of bacterial targets. However, IAC performance guidelines for nucleic acid diagnostics only requires IAC detection in the event of respective targets not being detected [27].

The multiplex capability of TEC-LAMP to simultaneously detect multiple bacterial meningitis pathogens is essential as approximately 1% of all cases are a result of co-infection, such as *S. pneumoniae* with *N. meningitidis* or *S. pneumoniae* with *H. influenzae* [11]. The TEC-LAMP assay successfully demonstrated the simultaneous detection of all three bacterial pathogens in the presence of 50 copies IAC template (Figure 2A–C, blue). In this reaction, the IAC was not detected (Figure 2D, blue) due to inhibition from co-amplification of the three bacterial targets. However, this is the result of the IAC template being present at a low copy number (50 copies), and the IAC TEC-LAMP oligonucleotides being present at lower concentrations relative to the bacterial target TEC-LAMP oligonucleotides, to favor bacterial target detection. Both control reactions in this experiment performed successfully. The IAC template was detected in the absence of bacterial targets (Figure 2D, red) indicating a successful uninhibited reaction. Also, the no template control (NTC) reaction, incorporating molecular grade water in place of bacterial or IAC templates, performed successfully (Figure 2A–D, black) as no amplification was observed. This result demonstrated that the TEC-LAMP assay does not produce false positive results via oligonucleotide cross-reactivity in the absence of bacterial or IAC templates.

Performance or cost limitations of existing bacterial meningitis diagnostic methods has created demand for a rapid, sensitive, specific, low-cost, multiplex molecular diagnostic technology. To address this demand, multiplex LAMP methodologies have been reported; however, all approaches to date possess various limitations. Restriction enzyme gel electrophoresis [34], lateral flow biosensors [35] and pyrosequencing methods [36] have been used to demonstrate multiplex LAMP technology. These approaches however require laborious, contamination-prone, post-amplification analysis, preventing real-time quantifiable detection [37]. Multiplex LAMP based on antibody-antigen interactions has also been developed using immuno-chromatographic strips [38]. This approach however is difficult for non-culturable organisms and dependent on unique pathogen surface antigens. Turbidity monitoring [39] to achieve multiplex LAMP has also been reported; however, this approach has poor reproducibility and requires extensive design optimization to achieve differentiable time-to-positivity values.

Various closed-system multiplex LAMP methodologies have been reported. Tanner and colleagues developed a detection of amplification by release of quenching (DARQ) LAMP technology utilizing strand displacement of a quencher labeled inner primer hybridized to a shorter fluorophore labeled oligonucleotide [40]. This technology however inhibits the standard LAMP reaction, and is associated with non-template amplification and reduced time-to-positivity in multiplex format, major drawbacks for point-of-care (POC) or clinical applications. DARQ LAMP is also identical to a previously reported duplex LAMP method using “assimilating probe” technology [41]. This method however reported poor analytical sensitivity in both singleplex and duplex formats. QUASR LAMP [24,25] is a multiplex LAMP technology with the converse labeling arrangement of DARQ LAMP, utilizing displacement of a fluorophore labeled inner primer hybridized to a shorter quencher labeled oligonucleotide. The fluorophore labeled inner primer is incorporated into the LAMP product and excess quencher labeled oligonucleotide suppresses any fluorescence from unincorporated inner primer. However, QUASR LAMP is limited to end-point detection and has only demonstrated duplex LAMP reactions. Also, due to the requirement of “excess” quencher labeled oligonucleotide, significantly increased oligonucleotide is required for each target compared to standard LAMP, thus increasing the possibility of non-specific detection. Wang and colleagues reported MERT-LAMP, a multiplex technology using Nb.*BsrDI* endonuclease and an inner primer containing the Nb.*BsrDI* restriction site, flanked by a fluorophore and quencher [42]. This method however possesses a design limitation requiring the Nb.*BsrDI* restriction site to be absent from the diagnostic target to prevent unspecific cleavage disrupting the LAMP cycle. Also, addition of this restriction site sequence onto the inner primer significantly affects standard LAMP assay design, as well as Tm and G/C content values, increasing potential for inhibitory secondary structure formation. 

Recently, Dou and colleagues detailed two separate multiplex LAMP methods for the detection of *S. pneumoniae*, *N. meningitidis* and *H. influenzae* through the use of polymer/paper hybrid biochips/spinchips [23,43]. These methods however do not enable real-time detection, require expensive fluorescence microscopy to detect low-level target concentrations and do not enable internal control incorporation directly into the LAMP reaction chambers. Also, the NTC reactions reported by Dou and colleagues did not incorporate the LAMP primers present in the target reactions, presenting possible issues with reproducing effective signal differentiation between target and NTC reactions.

The only TEC-LAMP design limitation is requirement of a thymine residue, for quencher placement, to be in close proximity to the 5′ fluorophore for sufficient quenching. This limitation however, can be overcome by coordinating the quencher to the adjacent sugar-phosphate primer backbone, instead of the thymine residue. TEC-LAMP does not create any additional design considerations to the standard LAMP method. Also, TEC-LAMP modifications cause minimal inhibition to the standard LAMP reaction (Supplemental Figure S1) indicating that incorporation of *Tth* endonuclease IV and the TEC primer/probe does not interfere with typical LAMP conditions. This also establishes that the cleaved 5′ end of the TEC primer/probe can still hybridize to its complement (Figure 1F), maintaining loop structure formation capabilities after cleavage and confirming primer/probe properties. The multiplex TEC-LAMP method uses biased non-equimolar primer set concentrations, providing simplified assay optimization through varying the TEC-LAMP oligonucleotide mix volumes added to the final reaction. Optimization of these primer set mix volumes involves initially testing all sets at the same volume and subsequently adjusting these volumes based on assay performance, increased volumes for sub-optimal performing primer sets and decreased volumes for optimal performing primer sets. Relative inner, outer and loop primer concentration ratios remain constant between primer sets, with a 50/50 ratio between the TEC primer/probe and forward inner primer. We observed that this 50/50 TEC primer/probe and forward inner primer ratio produced optimal results, while also reducing costs of using the more expensive TEC primer/probe as the only forward inner primer component. TEC-LAMP was evaluated on real-time PCR instrumentation; however this technology is compatible with cheaper multi-channel thermostatic fluorometers such as the Genie III^®^ (OptiGene Ltd., Horsham, UK) or Twista^®^ (TwistDx Ltd., Cambridge, UK). TEC-LAMP can also be applied to existing POC technology [21,22,23,24,25], such as simple heating devices combined with basic fluorescent detection methods.

The TEC-LAMP assay introduced in this study is the first report of a single-tube internally controlled multiplex LAMP assay with clinically relevant analytical sensitivity, and the first clinical evaluation of such technology applied to leading bacterial meningitis causative pathogens: *S. pneumoniae*, *N. meningitidis* and *H. influenzae*. This is also the first report of *Tth* endonuclease IV incorporation into nucleic acid amplification diagnostic technology. The TEC-LAMP methodology detailed is this study contributes to the current state-of-the-art in nucleic acid amplification diagnostics, providing novel transferable technology for infectious disease POC testing in low-resource disease-burdened areas.

## 4. Materials and Methods

### 4.1. Bacterial Strains, DNA Isolation and Quantification

A total of 168 bacterial reference strains and clinical isolates were evaluated in this study (Supplemental Table S1). Clinical isolates were collected from culture-confirmed cases of bloodstream infection as part of routine diagnostic service at Galway University Hospital (GUH). All strains, stored at −80 °C, were cultured in brain heart infusion (BHI) media (Oxoid, Hampshire, UK) and incubated at 37 °C for 18 h, under microaerophilic conditions, excluding *Haemophilus* strains which were cultured using Haemophilus test media (Oxoid). Genomic DNA was extracted using the DNeasy Blood and Tissue kit (Qiagen, Hilden, Germany) and quantified using the Qubit dsDNA broad range/high sensitivity assay kits and Qubit 2.0 fluorometer (Life Technologies, Warrington, UK), according to manufacturer’s instructions. Resulting DNA concentrations were converted to genome copy values using genome size standards of 2.1 Mb, 2.2 Mb and 1.83 Mb for *S. pneumoniae*, *N. meningitidis* and *H. influenzae*, respectively.

### 4.2. Diagnostic Targets and TEC-LAMP Oligonucleotides

*SPNA45_01710*, a chromosomal heparinise III-like protein gene (position 1743404 to –1743847 of accession number HE983624.1), and *NMO_1242*, a chromosomal cytolysin secretion ABC transporter gene (position 1295659 to 1297887 of accession number AM889136.1), were identified as novel diagnostic targets for *S. pneumoniae* and *N. meningitidis*, respectively. Alignment analysis of whole genome sequences retrieved from the National Center for Biotechnology Information (NCBI) database identified these biomarkers as highly conserved orthologous regions. The *pstA* gene was previously identified as an effective diagnostic target for *H. influenzae* [32], and the IAC template was a 500 bp random DNA gBlocks^®^ Gene Fragment (Supplemental Table S4) purchased from Integrated DNA Technologies (Leuven, Belgium). These targets were used to design TEC-LAMP oligonucleotides (Table 1) using PrimerExplorer V4 (Eiken Chemical, Tokyo, Japan). Standard desalted oligonucleotide primers were synthesized by Integrated DNA Technologies. TEC primer/probes for *S. pneumoniae*, *N. meningitidis*, *H. influenzae* and the IAC, labeled with FAM, Cy5, HEX and Cyan fluorophores, respectively, were HPLC purified and synthesized by Metabion International AG (Planegg, Germany). Each fluorophore corresponded to one of four detection channels of the LightCycler^®^ 480 instrument II (Roche Diagnostics, Sussex, UK) used to perform the LAMP reactions.

### 4.3. Internally Controlled Multiplex TEC-LAMP Assay

TEC-LAMP oligonucleotide mixes consisting of 20 µM reverse inner, 10 µM forward inner and TEC primer/probe, 5 µM forward and reverse loop, and 2.5 µM forward and reverse outer, were prepared for each target and varying volumes were added to the final multiplex TEC-LAMP reaction: 3.25 µL *S. pneumoniae*, 1.25 µL *N. meningitidis*, 1.5 µL *H. influenzae* and 0.75 µL IAC. The final TEC-LAMP reaction contained 1× Isothermal Amplification Buffer (New England Biolabs, Hitchin, UK), 6 mM MgSO_4_ (Roche Diagnostics), 1.4 mM deoxynucleotide triphosphate set (New England Biolabs), *S. pneumoniae* oligonucleotides [2.6 µM reverse inner, 1.3 µM forward inner and TEC primer/probe, 0.65 µM forward and reverse loop, 0.325 µM forward and reverse outer], N. meningitis oligonucleotides [1 µM reverse inner, 0.5 µM forward inner and TEC primer/probe, 0.25 µM forward and reverse loop, 0.125 µM forward and reverse outer], H. influenzae oligonucleotides [1.2 µM reverse inner, 0.6 µM forward inner and TEC primer/probe, 0.3 µM forward and reverse loop, 0.15 µM forward and reverse outer], IAC oligonucleotides [0.6 µM reverse inner, 0.3 µM forward inner and TEC primer/probe, 0.15 µM forward and reverse loop, 0.075 µM forward and reverse outer], 8 U Bst 2.0 WarmStart DNA polymerase (New England Biolabs), 15 U *Tth* endonuclease IV (New England Biolabs), 1 µL IAC template (50 copies), 1 µL DNA template (1–3 templates) or 1 µL molecular grade water for no template control (NTC) reactions, and molecular grade water to give a final volume of 25 µL. Reactions were performed for 60 × 1 min cycles at 67 °C in a LightCycler^®^ 480 instrument II (Roche Diagnostics). The fluorescence detection channels used were 450–500 nm (Cyan), 495–520 nm (FAM), 535–565 nm (HEX) and 646–662 nm (Cy5), with fluorescent measurements recorded every cycle. A color compensation file, generated as per LightCycler^®^ 480 operator manual, was applied for correction of any channel-to-channel fluorescence cross-talk.

### 4.4. Demonstration of Internally Controlled Multiplex TEC-LAMP Detection

For demonstration of the multiplex TEC-LAMP assay, type strain purified genomic DNA templates (*S. pneumoniae* DSM 20566, *N. meningitidis* NCTC 10025 and *H. influenzae* DSM 4690) were tested at 100 genome copy concentrations. Internally controlled multiplex TEC-LAMP detection was demonstrated by challenging the assay with a combination of all three templates, in the presence of 50 copies IAC template. Control reactions with no bacterial template in the presence of 50 copies IAC template, and a NTC reaction, were performed in parallel. The LightCycler^®^ 480 recorded positive results as exponential signal acquisition exceeding background fluorescence, represented as fluorescence amplification curves (Figure 2). Cycle threshold (Ct) values denoted cycles at which fluorescent signal exceeded background levels. As TEC-LAMP reactions were performed for 60 × 1 min cycles on the LightCycler^®^ 480 instrument II, resulting Ct-values acted as approximate time-to-positivity values in minutes.

### 4.5. Analytical Specificity and Limit of Detection

The analytical specificity of the TEC-LAMP assay was established by testing purified genomic DNA from a panel of bacterial strains (Supplemental Table S1) at 10^5^ genome copy concentrations. The TEC-LAMP assay limit of detection (LOD) for *S. pneumoniae*, *N. meningitidis* or *H. influenzae*, in the presence of 50 copies IAC template, was established with 95% probability using Probit regression analysis. Type strain purified genomic DNA for each pathogen was tested using 6 replicates of 128, 64, 32, 16, 8 and 4 genome copy concentrations. Probit analysis was performed on the resulting data (Supplemental Table S2) using Minitab 17 (MiniTab, State College, PA, USA).

### 4.6. Clinical Evaluation

TEC-LAMP clinical performance was evaluated by testing archived genomic DNA from PCR-confirmed cases of invasive pneumococcal, meningococcal and Haemophilus infection. All samples evaluated were supplied by the Irish Meningitis and Sepsis Reference Laboratory (IMSRL), Dublin, Ireland. The IMSRL extracted genomic DNA from anonymized residual clinical specimens, followed by real-time PCR analysis for *S. pneumoniae*, *N. meningitidis* and *H. influenzae*, as part of routine diagnostic service. IMSRL DNA extractions were carried out in 210 µL volumes using a QIAsymphony SP/AS instrument with QIAamp DSP DNA Blood Mini Kits (Qiagen), as per manufacturer instructions, for automated extractions in 50 µL elution volumes. IMSRL PCR assays (Supplemental Table S5) were performed using standard reaction conditions targeting the *S. pneumoniae lytA*, *N. meningitidis ctrA* and *H. influenzae fucK* genes, testing 2.5 µL of each sample. A total of 65 samples including 34 blood, 5 blood culture, 17 CSF, 5 pleural fluid, 1 knee fluid and 3 other body fluids, were supplied with corresponding real-time PCR Ct-values (Supplemental Table S3). For comparative purposes, the diagnostic sensitivity and specificity of the TEC-LAMP assay was also determined by testing 2.5 µL of each of sample. Any samples not detected using the TEC-LAMP assay were re-tested with “in-house” singleplex real-time PCR assays (Supplemental Table S6) using standard reaction conditions, targeting the *S. pneumoniae lepA*, *N. meningitidis NMO_1242* and *H. influenzae pstA* genes. 

## Figures and Tables

**Table 1:** TEC-LAMP oligonucleotides.

Primer Type	Sequence (5′-3′)
***S. pneumoniae***	
TEC primer/probe	(FAM)TGGA(dSpacer)AA(BHQ1-dT)GCTCTGGCTTTTGAAGTGA-CCTACACCAATATCCTCGCT
Forward Inner	TGGAAAATGCTCTGGCTTTTGAAGTGA-CCTACACCAATATCCTCGCT
Reverse Inner	TCTGTCTGGTAGACAGAATGACGGA-TCTTTGAGAATCAGATGCTGGA
Forward Outer	TCCGTCAACGAGGCACAA
Reverse Outer	AGCAAACTCACCAAGCGC
Forward Loop	TGATGAAACAGACAAGCTGATTCT
Reverse Loop	GCGCAATGATGGTATAATCCAG
***N. meningitidis***	
TEC primer/probe	(Cy5)TGTC(dSpacer)G(BHQ2-dT)GGCTTTGTTGGTGGTGTCGC-GTGCAAACAGATACGTCCG
Forward Inner	TGTCGGTGGCTTTGTTGGTGGTGTCGC-GTGCAAACAGATACGTCCG
Reverse Inner	CCGATGTACCAGCACCTTGTCC-GTTTGCGCTGATTACGCCTC
Forward Outer	CCCAATTCCACATCAATACGTG
Reverse Outer	GTGGTGTCGGTGGTGTTG
Forward Loop	GAGATTGTGTTGGGCGGTTTG
Reverse Loop	CACCACTTGGAAAAACAGAGGC
***H. influenzae***	
TEC primer/probe	(HEX)TGCC(dSpacer)C(BHQ1-dT)GCTTCACGTAAATTATTTGG-TGCTTATTCCTATCGTGGTACG
Forward Inner	TGCCGCTGCTTCACGTAAATTATTTGG-TGCTTATTCCTATCGTGGTACG
Reverse Inner	CTTGGTTGCTCTCAATGGCAAG-GCACGCCAGTTAAAATCCCT
Forward Outer	GGCTGGAGCATTCGCATT
Reverse Outer	TTCTCCTGAAATTCGGGCAA
Forward Loop	AACATATTGTCCGTAGTGCG
Reverse Loop	TGATGATTTGTTATCGAGCAGC
**Internal Amplification Control**	
TEC primer/probe	(CYAN)TGTT(dSpacer)A(BHQ1-dT)ATCCGCGATCCTTGCGTTGT-TCCCCGCTATGGAAGGTC
Forward Inner	TGTTTATATCCGCGATCCTTGCGTTGT-TCCCCGCTATGGAAGGTC
Reverse Inner	CACCTGTTCGTGTCGTATCGGT-ATGCATTACCAGAGTGCTCC
Forward Outer	TACAGCGAAAAGCCCAGC
Reverse Outer	AAGCGACGAATGTCCTGTG
Forward Loop	TCTTAATTGCTTGCCGGAGC
Reverse Loop	GAGCCATGTGCCATACTCGTC

FAM, 6-carboxyfluorescein fluorophore; dSpacer, 1′,2′-dideoxyribose; BHQ1-dT, black hole quencher 1 linked to thymine; -, separation between 5′ antisense and 3′ sense inner primer sequences; Cy5, cyanine fluorophore; BHQ2-dT, black hole quencher 2 linked to thymine; HEX, 6-hexachlorofluorescein fluorophore; CYAN, LC^®^CYAN 500 fluorophore.

## Supplementary Materials

The following are available online at http://www.mdpi.com/1422-0067/19/2/524/s1.
