# Supplementary material for: Evaluation of an Internally Controlled Multiplex Tth Endonuclease Cleavage Loop-Mediated Isothermal Amplification (TEC-LAMP) Assay for the Detection of Bacterial Meningitis Pathogens
Source: Int J Mol Sci. 2018 Feb 9;19(2):524. doi: 10.3390/ijms19020524 (PMC5855746; doi:10.3390/ijms19020524)
Supplement: Supplementary file 1 [file ijms-19-00524-s001.docx]

**Supplemental Data**

| **Table S1:** **Internally controlled multiplex TEC-LAMP assay specificity panel.** | | | | | |
| --- | --- | --- | --- | --- | --- |
| Organism | Strain | TEC-LAMP Result | | | |
|  |  | *S. pneumoniae* (FAM) | *N. meningitidis* (Cy5) | *H. influenzae* (HEX) | IAC  (Cyan) |
| **Inclusivity**  **Panel** | | | | | |
| *Streptococcus pneumoniae* Reference Strains and Clinical Isolates | | | | | |
| *S. pneumoniae* (1, Type strain) | DSM 20566 | + | - | - | - |
| *S. pneumoniae* (3) | DSM 14377 | + | - | - | - |
| *S. pneumoniae* (5) | DSM 14378 | + | - | - | - |
| *S. pneumoniae* (9V) | DSM 11865 | + | - | - | - |
| *S. pneumoniae* (19F) | DSM 24048 | + | - | - | - |
| *S. pneumoniae* (19F) | DSM 25971 | + | - | - | - |
| *S. pneumoniae* (23F) | DSM 11866 | + | - | - | - |
| *S. pneumoniae* (23F) | DSM 11868 | + | - | - | - |
| Clinical Isolates | 14 Isolates | + | - | - | - |
| *Neisseria meningitidis* Reference Strains and Clinical Isolates | | | | | |
| *N. meningitidis* (A, type strain) | NCTC 10025 | - | + | - | - |
| *N. meningitidis* (A) | DSM 10036 | - | + | - | - |
| *N. meningitidis* (A) | NCTC 3372 | - | + | - | - |
| *N. meningitidis* (A) | NCTC 3375 | - | + | - | - |
| *N. meningitidis* (B) | ATCC 13090 | - | + | - | - |
| *N. meningitidis* (C) | ATCC 13102 | - | + | - | - |
| *N. meningitidis* (C) | DSM 15464 | - | + | - | - |
| *N. meningitidis* (W) | NCTC 11203 | - | + | - | - |
| *N. meningitidis* (X) | NCTC 10790 | - | + | - | - |
| *N. meningitidis* (Y) | NCTC 10791 | - | + | - | - |
| Clinical Isolates | 9 Isolates | - | + | - | - |
| *Haemophilus influenzae* Reference Strains and Clinical Isolates | | | | | |
| *H. influenzae* (b, Type Strain) | DSM 4690 | - | - | + | - |
| *H. influenzae* (a) | NCTC 8465 | - | - | + | - |
| *H. influenzae* (b) | DSM 10001 | - | - | + | - |
| *H. influenzae* (b) | DSM 11969 | - | - | + | - |
| *H. influenzae* (b) | DSM 11970 | - | - | + | - |
| *H. influenzae* (b) | DSM 24049 | - | - | + | - |
| *H. influenzae* (c) | NCTC 8469 | - | - | + | - |
| *H. influenzae* (d) | DSM 11121 | - | - | + | - |
| *H. influenzae* (e) | NCTC 8472 | - | - | + | - |
| *H. influenzae* (f) | DSM 10000 | - | - | + | - |
| *H. influenzae* (aegyptius) | DSM 21187 | - | - | + | - |
| Clinical Isolates | 19 Isolates | - | - | + | - |
| **Exclusivity**  **Panel** | | | | | |
| *Streptococcus* Reference Strains (excl. *S. pneumoniae*) | | | | | |
| *S. agalactiae* | BCCM 15081 | - | - | - | + |
| *S. agalactiae* | BCCM 15082 | - | - | - | + |
| *S. agalactiae* | BCCM 15083 | - | - | - | + |
| *S. agalactiae* | BCCM 15084 | - | - | - | + |
| *S. agalactiae* | BCCM 15085 | - | - | - | + |
| *S. agalactiae* | BCCM 15086 | - | - | - | + |
| *S. agalactiae* | BCCM 15087 | - | - | - | + |
| *S. agalactiae* | BCCM 15094 | - | - | - | + |
| *S. agalactiae* | BCCM 15095 | - | - | - | + |
| *S. anginosus* | DSM 20563 | - | - | - | + |
| *S. australis* | DSM 15627 | - | - | - | + |
| *S. bovis* | DSM 20480 | - | - | - | + |
| *S. canis* | DSM 20715 | - | - | - | + |
| *S. constellatus* | DSM 20575 | - | - | - | + |
| *S. cristatus* | DSM 8249 | - | - | - | + |
| *S. downei* | DSM 5635 | - | - | - | + |
| *S. dysgalactiae subsp. equisimilis* | DSM 6176 | - | - | - | + |
| *S. equi subsp. equi* | DSM 20561 | - | - | - | + |
| *S. equinus* | DSM 20558 | - | - | - | + |
| *S. gordonii* | DSM 6777 | - | - | - | + |
| *S. infantis* | DSM 12492 | - | - | - | + |
| *S. intermedius* | DSM 20573 | - | - | - | + |
| *S. mitis* | DSM 12643 | - | - | - | + |
| *S. mutans* | DSM 20523 | - | - | - | + |
| *S. oralis* | DSM 20066 | - | - | - | + |
| *S. parasanguinis* | DSM 6778 | - | - | - | + |
| *S. peroris* | DSM 12493 | - | - | - | + |
| *S. porcinus* | DSM 20725 | - | - | - | + |
| *S. pseudopneumoniae* | DSM 18670 | - | - | - | + |
| *S. pyogenes* | DSM 2072 | - | - | - | + |
| *S. pyogenes* | DSM 20565 | - | - | - | + |
| *S. salivarius* | DSM 20560 | - | - | - | + |
| *S. salivarius* | DSM 20617 | - | - | - | + |
| *S. sanguinis* | DSM 20567 | - | - | - | + |
| *S. sinensis* | DSM 14990 | - | - | - | + |
| *S. suis* | DSM 9682 | - | - | - | + |
| *S. uberis* | DSM 20569 | - | - | - | + |
| *S. vestibularis* | DSM 5636 | - | - | - | + |
| *Neisseria* Reference Strains (excl. *N. meningitidis*) | | | | | |
| *N. animalis* | DSM 23392 | - | - | - | + |
| *N. animaloris* | DSM 21642 | - | - | - | + |
| *N. bacilliformis* | DSM 23338 | - | - | - | + |
| *N. canis* | DSM 18000 | - | - | - | + |
| *N. caviae* | DSM 23336 | - | - | - | + |
| *N. cuniculi* | DSM 21768 | - | - | - | + |
| *N. dentiae* | DSM 19151 | - | - | - | + |
| *N. elongata subsp. elongata* | DSM 17712 | - | - | - | + |
| *N. elongata subsp. glycolytica* | DSM 23337 | - | - | - | + |
| *N. elongata subsp. nitroreducens* | DSM 17632 | - | - | - | + |
| *N. flavescens* | DSM 17633 | - | - | - | + |
| *N. gonorrhoeae* | ATCC 19424 | - | - | - | + |
| *N. gonorrhoeae* | DSM 9188 | - | - | - | + |
| *N. gonorrhoeae* | DSM 9189 | - | - | - | + |
| *N. lactamica* | ATCC 23970 | - | - | - | + |
| *N. lactamica* | DSM 4691 | - | - | - | + |
| *N. macacae* | DSM 19175 | - | - | - | + |
| *N. mucosa* | DSM 17611 | - | - | - | + |
| *N. ovis* | DSM 18075 | - | - | - | + |
| *N. perflava* | DSM 18009 | - | - | - | + |
| *N. polysaccharea* | DSM 22809 | - | - | - | + |
| *N. shayeganii* | DSM 22246 | - | - | - | + |
| *N. sicca* | DSM 17713 | - | - | - | + |
| *N. subflava* | DSM 17610 | - | - | - | + |
| *N. wadsworthii* | DSM 22247 | - | - | - | + |
| *N. weaveri* | DSM 17688 | - | - | - | + |
| *N. zoodegmatis* | DSM 21483 | - | - | - | + |
| *N. zoodegmatis* | DSM 21643 | - | - | - | + |
| *Haemophilus* Reference Strains (excl. *H. influenzae*) | | | | | |
| *H. actinomycetemcomitans* | DSM 8324 | - | - | - | + |
| *H. actinomycetemcomitans* | DSM 11122 | - | - | - | + |
| *H. aphrophilus* | NCTC 11096 | - | - | - | + |
| *H. avium* | DSM 18557 | - | - | - | + |
| *H. ducreyi* | DSM 8925 | - | - | - | + |
| *H. ducreyi* | NCTC 11479 | - | - | - | + |
| *H. equigenitalis* | DSM 10668 | - | - | - | + |
| *H. felis* | DSM 21192 | - | - | - | + |
| *H. haemoglobinophilus* | DSM 21241 | - | - | - | + |
| *H. haemolyticus* | CCUG 12834 | - | - | - | + |
| *H. haemolyticus* | CCUG 15312 | - | - | - | + |
| *H. haemolyticus* | CCUG 15642 | - | - | - | + |
| *H. haemolyticus* | CCUG 24149 | - | - | - | + |
| *H. haemolyticus* | CCUG 34110 | - | - | - | + |
| *H. haemolyticus* | CCUG 36015 | - | - | - | + |
| *H. haemolyticus* | CCUG 36016 | - | - | - | + |
| *H. haemolyticus* | CDC-M19501 | - | - | - | + |
| *H. haemolyticus* | CDC-M21127 | - | - | - | + |
| *H. haemolyticus* | CDC-M21621 | - | - | - | + |
| *H. haemolyticus* | NCTC 10839 | - | - | - | + |
| *H. paracuniculus* | DSM 21452 | - | - | - | + |
| *H. paragallinarum* | DSM 18554 | - | - | - | + |
| *H. parahaemolyticus* | DSM 21417 | - | - | - | + |
| *H. parainfluenzae* | DSM 8978 | - | - | - | + |
| *H. paraphrohaemolyticus* | DSM 21451 | - | - | - | + |
| *H. parasuis* | DSM 21448 | - | - | - | + |
| *H. pittmaniae* | DSM 17240 | - | - | - | + |
| *H. pittmaniae* | DSM 21203 | - | - | - | + |
| *H. segnis* | NCTC 10977 | - | - | - | + |
| *H. somnus* | CCUG 12839 | - | - | - | + |
| *H. vaginalis* | DSM 4944 | - | - | - | + |
| DSM, Leibniz Institute DSMZ - German Collection of Microorganisms and Cell Cultures; BCCM, Belgian Coordinated Collections of Microorganisms; NCTC, National Collection of Type Cultures; CCUG, Culture Collection, University of Göteborg, Sweden; CDC, Centre for Disease Control; ATCC, American Type Culture Collection; +, positive; -, negative. | | | | | |

| **Table S2: Limit of detection (LOD) Probit analysis for the internally controlled multiplex TEC-LAMP detection of *S. pneumoniae*, *N. meningitides* or *H. influenzae* in the presence of 50 copies IAC template.** | | | |
| --- | --- | --- | --- |
| Genome copy concentration tested | Replicates detected / Replicates tested | | |
|  | *S. pneumoniae* | *N. meningitidis* | *H. influenzae* |
| 128 | 6 / 6 | 6 / 6 | 6 / 6 |
| 64 | 6 / 6 | 6 / 6 | 6 / 6 |
| 32 | 5 / 6 | 6 / 6 | 6 / 6 |
| 16 | 4 / 6 | 6 / 6 | 4 / 6 |
| 8 | 1 / 6 | 2 / 6 | 2 / 6 |
| 4 | 0 / 6 | 1 / 6 | 0 / 6 |
| **Genome copy LOD per reaction**  **(95% probability)** | **39.5** | **17.3** | **25.9** |

| **Table S3: Internally controlled multiplex TEC-LAMP assay clinical samples tested.** | | | | | | | |
| --- | --- | --- | --- | --- | --- | --- | --- |
| Sample  No. | Clinical Specimen | IMSRL PCR  (Ct Value) | TEC-LAMP Result | | | | |
|  |  |  | TEC-LAMP  (Ct Value) | *S. pneumoniae* (FAM) | *N. meningitidis* (Cy5) | *H. influenzae* (HEX) | IAC (Cyan) |
| ***S. pneumoniae* Clinical Samples** | | | | | | | |
| 1 | BLD | 34.04 | 17.94 | + | - | - | + |
| 2 | BLD | 31.88 | 22.54 | + | - | - | + |
| 3 | BLD | 30.17 | 20.08 | + | - | - | + |
| 4 | BLD | 27.16 | 25.40 | + | - | - | + |
| 5 | BLD | 25.30 | 39.57 | + | - | - | + |
| 6 | BLD | 36.11 | 53.32 | + | - | - | + |
| 7 | CSF | 23.23 | 24.61 | + | - | - | + |
| 8 | CSF | 36.96 | - | - | - | - | + |
| 9 | CSF | 27.95 | 14.27 | + | - | - | - |
| 10 | CSF | 33.92 | 21.42 | + | - | - | + |
| 11 | CSF | 33.65 | 23.54 | + | - | - | + |
| 12 | CSF | 35.81 | 27.17 | + | - | - | + |
| 13 | CSF | 26.64 | 15.59 | + | - | - | - |
| 14 | CSF | 29.67 | 22.59 | + | - | - | + |
| 15 | CSF | 25.67 | 17.13 | + | - | - | - |
| 16 | FLD | 32.71 | 21.91 | + | - | - | + |
| 17 | FLD | 27.61 | 18.20 | + | - | - | - |
| 18 | FLD | 30.55 | 26.53 | + | - | - | + |
| 19 | PLF | 34.02 | 18.80 | + | - | - | + |
| 20 | PLF | 29.75 | 21.74 | + | - | - | + |
| 21 | PLF | 28.83 | 18.97 | + | - | - | + |
| 22 | PLF | 34.36 | 18.71 | + | - | - | + |
| 23 | Knee FLD | 25.27 | 16.29 | + | - | - | - |
| ***N. meningitidis* Clinical Samples** | | | | | | | |
| 24 | BLD | 36.93 | 21.45 | - | + | - | + |
| 25 | BLD | 29.69 | 13.63 | - | + | - | - |
| 26 | BLD | 29.42 | 16.01 | - | + | - | + |
| 27 | BLD | 28.37 | 11.14 | - | + | - | - |
| 28 | BLD | 30.14 | 15.21 | - | + | - | - |
| 29 | BLD | 35.79 | 20.09 | - | + | - | + |
| 30 | BLD | 28.11 | 11.14 | - | + | - | - |
| 31 | BLD | 37.10 | 20.51 | - | + | - | + |
| 32 | BLD | 33.04 | 22.75 | - | + | - | + |
| 33 | BLD | 35.08 | 18.59 | - | + | - | + |
| 34 | BLD | 36.57 | 20.70 | - | + | - | + |
| 35 | BLD | 35.77 | 22.70 | - | + | - | + |
| 36 | BLD | 23.41 | 11.24 | - | + | - | - |
| 37 | BLD | 34.71 | 20.58 | - | + | - | + |
| 38 | BLD | 30.84 | 18.75 | - | + | - | + |
| 39 | BLD | 34.03 | 13.85 | - | + | - | + |
| 40 | BLD | 34.08 | 19.63 | - | + | - | + |
| 41 | BLD | 38.67 | 34.18 | - | + | - | + |
| 42 | CSF | 38.27 | 33.23 | - | + | - | + |
| 43 | CSF | 35.00 | 35.85 | - | + | - | + |
| 44 | CSF | 27.61 | 9.56 | - | + | - | - |
| 45 | CSF | 28.09 | 9.95 | - | + | - | - |
| ***H. influenzae* Clinical Samples** | | | | | | | |
| 46 | BLD | 38.21 | - | - | - | - | + |
| 47 | BLD | 35.89 | 55.00 | - | - | + | + |
| 48 | BLD | 38.30 | - | - | - | - | + |
| 49 | BLD | 37.56 | 24.55 | - | - | + | + |
| 50 | BLD | 36.32 | 21.19 | - | - | + | + |
| 51 | BLD | 37.77 | 27.28 | - | - | + | + |
| 52 | BLD | 33.84 | 23.80 | - | - | + | + |
| 53 | BLD | 39.78 | - | - | - | - | + |
| 54 | BLD | 39.62 | - | - | - | - | + |
| 55 | BLD | 34.21 | 21.35 | - | - | + | + |
| 56 | CSF | 37.36 | 23.30 | - | - | + | + |
| 57 | CSF | 32.95 | 28.38 | - | - | + | + |
| 58 | CSF | 22.17 | 12.61 | - | - | + | - |
| 59 | CSF | 24.99 | 12.60 | - | - | + | - |
| 60 | BC | 16.55 | 13.01 | - | - | + | - |
| 61 | BC | 16.72 | 15.15 | - | - | + | - |
| 62 | BC | 15.51 | 12.95 | - | - | + | - |
| 63 | BC | 17.59 | 12.93 | - | - | + | - |
| 64 | BC | 15.06 | 13.99 | - | - | + | - |
| 65 | PLF | 22.36 | 12.00 | - | - | + | - |
| BLD, blood; CSF, cerebrospinal fluid; FLD, fluid; PLF, pleural fluid; BC, blood culture; +, positive; -, negative. | | | | | | | |

| **Table S4. Internal amplification control (IAC) gBlocks® Gene Fragment nucleotide sequence (5’ - 3’).** |
| --- |
| CGGCGCAGTGCTGCCGACAGCCGGGCATTGTCTTTGGGGCGTTATTCGAGGGCACCCGGACCTAACTTGTCGGGACCACCC  GGGGTAGTCATCGGGCTTATACAGCGAAAAGCCCAGCACCCGGCTCCCCGCTATGGAAGGTCATTAGCTCCGGCAAGCAAT  TAAGAACAACGCAAGGATCGCGGATATAAACAGAGAAACGGCCGAATACACCTGTTCGTGTCGTATCGGTAAATAGCCTCG  CGGAGCCATGTGCCATACTCGTCTGCGGAGCACTCTGGTAATGCATATGGTCCACAGGACATTCGTCGCTTCCGGGTATGC  GCTCTATGTGACGGTCTTTTGGCGCACAAATGCTCAGCACCATTTAAATTAGACCGACTCCAGATCTGTAAGGTCCGCCAC  GCAGACGACAGCCCACGGAGACCACTGACCGATCTACCTGAACGGCGACCATCTGTGTGGTACTGGGGCGGAGAGATAACT  ACGGTGCCGCTTAC |

| **Table S5: IMSRL PCR oligonucleotides.** | |
| --- | --- |
| **Type** | **Sequence** |
| ***S. pneumoniae*** | |
| Forward | 5’-ACGCAATCTAGCAGATGAAGC-3’ |
| Probe | 5’-(FAM)CTCCCTGTATCAAGCGTTTTCGGCA(BHQ1)-3’ |
| Reverse | 5’-TCGTGCGTTTTAATTCCAGCT-3’ |
| ***N. meningitidis*** | |
| Forward | 5’-TTGTGTGGAAGTTTAATTGTAGGATGC-3’ |
| Probe | 5’-(FAM)TCCTTCATCAGGCCCCAGCG(BHQ1)-3’ |
| Reverse | 5’-TCAGATTGTTGCCCTAAAGAGACA-3’ |
| ***H. influenzae*** | |
| Forward | 5’-ATGGCGGGAACATCAATGA-3’ |
| Probe | 5’-(FAM)CGGTAATTGGGATCCAT(BHQ1)-3’ |
| Reverse | 5’-ACGCATAGGAGGGAAATGGTT-3’ |
| FAM, 6-carboxyfluorescein fluorophore; BHQ1, black hole quencher | |

| **Table S6: “In-house” PCR oligonucleotides.** | |
| --- | --- |
| **Type** | **Sequence** |
| ***S. pneumoniae*** | |
| Forward | 5’-CTCGTAAGCGTAAACTCCTTG-3’ |
| Probe | 5’-(FAM)ACGCATGAAATCCATCGGATCAGTT(BHQ1)-3’ |
| Reverse | 5’-CATACTCAAGACGCTGAGGA-3’ |
| ***N. meningitidis*** | |
| Forward | 5’-CGACATGTTCGAACGTAATCTCC-3’ |
| Probe | 5’-(FAM)TATCGGGCAAAGCCAAATGCGAAG(BHQ1)-3’ |
| Reverse | 5’-ATTTCGGTGGCGCGTTT-3’ |
| ***H. influenzae*** | |
| Forward | 5’-GGTACGCACYACGGACAATATG-3’ |
| Probe | 5’-(FAM)AGCTCTTGGTTGCTCTCAATGGCA(BHQ1)-3’ |
| Reverse | 5’-CCTGATTTAGCYGCTCGATAACA-3’ |
| FAM, 6-carboxyfluorescein fluorophore; BHQ1, black hole quencher | |

**
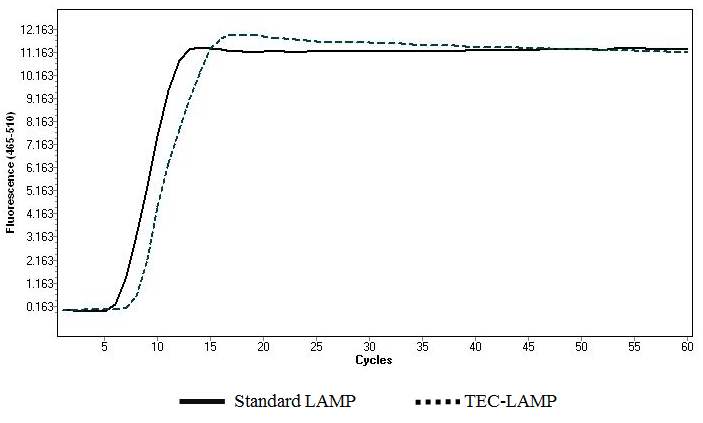
**

**Figure S1. Effect of TEC-LAMP modifications on standard LAMP reaction**. This experiment was carried out to determine if the TEC-LAMP modifications of oligonucleotide dye labels / abasic site and *Tth* endonuclease IV enzyme, inhibit the standard unmodified LAMP reaction. The TEC-LAMP reaction was performed as previously described, however, only the Cy5 labelled *N. meningitidis* primer set (Table 1) was used. For comparative purposes this reaction was performed with [dashed black] and without [solid black] the TEC-LAMP modifications. Both reactions were challenged with *N. meningitidis* DNA template at 10^4^ copies. Reactions were performed for 60 cycles of 67°C for 1 min using a LightCycler 480 instrument II, with fluorescence recorded at each cycle in the FAM (495-520 nm) detection channel. As the Cy5 dye label on the *N. meningitidis* primer set is not detected in the FAM (495-520 nm) detection channel, 0.1X SYBR Green 1 intercalating dye was used to monitor the reaction. The resulting amplification cures indicate that the TEC-LAMP modifications cause minimal reaction inhibition to the standard unmodified LAMP reaction with detection observed only 2 cycles later than the unmodified LAMP reaction.
